# Redox-Modulating Agents in the Treatment of Viral Infections

**DOI:** 10.3390/ijms21114084

**Published:** 2020-06-08

**Authors:** Paola Checconi, Marta De Angelis, Maria Elena Marcocci, Alessandra Fraternale, Mauro Magnani, Anna Teresa Palamara, Lucia Nencioni

**Affiliations:** 1 IRCCS San Raffaele Pisana, Department of Human Sciences and Promotion of the Quality of Life, San Raffaele Roma Open University, 00166 Rome, Italy; paola.checconi@uniroma5.it (P.C.); annateresa.palamara@uniroma1.it (A.T.P.); 2 Department of Public Health and Infectious Diseases, Laboratory Affiliated to Istituto Pasteur Italia-Fondazione Cenci Bolognetti, Sapienza University of Rome, 00185 Rome, Italy; marta.deangelis@uniroma1.it (M.D.A.); mariaelena.marcocci@uniroma1.it (M.E.M.); 3 Department of Biomolecular Sciences, University of Urbino Carlo Bo, 61029 Urbino (PU), Italy; alessandra.fraternale@uniurb.it (A.F.); mauro.magnani@uniurb.it (M.M.)

**Keywords:** redox state, reduced glutathione (GSH), antioxidants, Nrf2, viral infections, influenza, COVID-19

## Abstract

Viruses use cell machinery to replicate their genome and produce viral proteins. For this reason, several intracellular factors, including the redox state, might directly or indirectly affect the progression and outcome of viral infection. In physiological conditions, the redox balance between oxidant and antioxidant species is maintained by enzymatic and non-enzymatic systems, and it finely regulates several cell functions. Different viruses break this equilibrium and induce an oxidative stress that in turn facilitates specific steps of the virus lifecycle and activates an inflammatory response. In this context, many studies highlighted the importance of redox-sensitive pathways as novel cell-based targets for therapies aimed at blocking both viral replication and virus-induced inflammation. In the review, we discuss the most recent findings in this field. In particular, we describe the effects of natural or synthetic redox-modulating molecules in inhibiting DNA or RNA virus replication as well as inflammatory pathways. The importance of the antioxidant transcription factor Nrf2 is also discussed. Most of the data reported here are on influenza virus infection. We believe that this approach could be usefully applied to fight other acute respiratory viral infections characterized by a strong inflammatory response, like COVID-19.

## 1. Viral Infections and Redox State

In recent years, several studies have been focused on the role of redox-modulating agents in the treatment of viral infections. The aim of this research area is to identify new molecules able to target the host pathways exploited by viruses for their replication and/or to improve the immune response to viral infections [1,2]. In fact, viruses use several strategies to manipulate host cell machinery to their advantage, including modulation of the intracellular redox state. An imbalance of the redox state towards oxidant conditions is a key event during viral infections [3]. Our group showed for instance that influenza viruses induce oxidative stress mediated by an over-production of reactive oxygen species (ROS) and a decrease of reduced glutathione (GSH), the main intracellular antioxidant, and that such conditions favor viral replication [4,5,6]. The production of ROS is mediated by the activity of the nicotinamide adenine dinucleotide phosphate (NADPH) oxidase (NOX) family, which consists of seven members: NOX1 to NOX5 and the two dual oxidases, Duox1 and Duox2, expressed in most cell types [7]. Although NOX2 plays a crucial role in the killing of bacteria and fungi, it does not work in the same manner against viruses. For instance, NOX2 is produced by inflammatory cells in response to viral infections and enhances the pathology caused by viruses of low and high pathogenicity, including influenza A viruses [8]. Indeed, it has been shown that in the absence of NOX2, influenza virus causes less lung inflammation and injury, suggesting a critical role of this enzyme in control the infection [9]. NOX2 is expressed in endosomes following different types of infection and its expression is dependent on Toll-like Receptor-7 (TLR-7), that binds double stranded RNA (dsRNA), a transitory intermediate formed during the replication of many RNA viruses, and TLR-9, that recognizes unmethylated CpG motifs within viral genome of DNA viruses [10]. Furthermore, NOX2 activity suppresses antiviral signaling by the modification of a conserved single cysteine residue (Cys98) of TLR-7 [10]. In non-phagocytic cells, NOX2 generates ROS responsible for NF-κB activation during respiratory syncytial virus (RSV) and parainfluenza (Sendai) virus infections [11]. Another member of NOXs family, NOX4 has been described to be involved in controlling influenza virus replication. Indeed, this isoform is up-regulated following viral infection in lung epithelial cells, and it is responsible for the ROS generation [4]. NOX4-derived ROS activate p38 and ERK1-2 Mitogen activated protein kinases (MAPKs) that, in turn, promote the nuclear export of viral ribonucleoprotein favoring viral replication [12,13].

Interestingly, NOX4-derived ROS production has been shown to be modulated by Angiotensin-Converting Enzyme 2 (ACE2) [14], that is known to be the main receptor of severe acute respiratory syndrome-coronavirus (SARS-CoV) and now identified also as the key receptor for the novel SARS-CoV-2, cause of the recent respiratory syndrome COVID-19 [15].

The accumulation of ROS has been measured in many other types of viral infections. Hepatitis C virus (HCV) has been shown to promote oxidative stress and ROS production [16]. In particular, an intense oxidative stress induced by NOX4 activity with a decrease of GSH/GSSG ratio and an increase of apoptosis in infected cells has been described during the acute phase of HCV infection. Conversely, there was a recovery of reducing conditions during the chronic phase that was associated to viral persistence [17]. Interestingly, when oxidative stress was pharmacologically induced by treatment with Auranofin, a well-known pro-oxidant drug [18], viral RNA titer was significantly increased, thus suggesting that a pro-oxidant state may favor the reactivation of HCV replication. 

With regard to hepatitis B virus (HBV), a DNA virus, it has been reported that three viral proteins, the surface antigens HBx, HBsAg and the core antigen HBcAg, mediate ROS production [19,20,21]. However, further studies aimed at clarifying the mechanisms by which HBV induces oxidative stress and its role in viral replication are needed.

An accumulation of ROS has also been observed during human immunodeficiency virus (HIV) infection [22,23], mediated by the envelope protein gp120 [24] and Tat proteins [25]. Furthermore, NOX2 and NOX4-induced ROS overproduction has been reported in HIV gp120 treated astrocytes [26].

The level of oxidative stress is also critical for the immune response to viruses. In this context, it has been reported that oxidative stress observed during in vitro and in vivo Dengue virus (DENV) infections is important for the production of inflammatory cytokines [27]. In patients, the alteration in redox state has been correlated with disease severity [28]. Moreover, ROS production controls the antiviral and apoptotic programs in DENV-infected human monocyte derived dendritic cells (DC) [29]. 

In the redox state equilibrium, GSH is the main intracellular antioxidant that exerts an efficient buffering role against ROS, through the thiol group of its cysteine that oxidizes to the disulfide form (GSSG), then reduced back to the thiol form (GSH) by glutathione reductase. In addition to its ROS scavenger activity, GSH neutralizes other potentially harmful molecules such as metals and xenobiotics [30]. Moreover, it has a crucial role in cellular signaling and processes, including innate immune response to viruses [30,31,32]. During viral infections, an intracellular GSH depletion occurs through multiple mechanisms. It has been shown that Sendai virus, for instance, caused an early leak of GSH due to the perturbation of the cell membrane following to the virus fusion. Later in the viral cycle, GSH decrease was mainly due to the preferential incorporation of cysteines in viral proteins, while the formation of mixed disulfides between GSH and cellular proteins was also observed [33]. During influenza virus infection, our group found that GSH decrease was pivotal for the folding and maturation of viral glycoprotein haemagglutinin (HA) and therefore for viral replication [34].

In the balance of the intracellular redox state, the activation of nuclear factor erythroid 2-related factor 2 (Nrf2) plays a critical role. In fact, this transcription factor is basally maintained at low level in the cytosol by the adaptor protein Kelch-like ECH associated protein 1 (Keap1), that recruits Cul3-Rbx1 E3 ligase allowing Nrf2 ubiquitylation and subsequent proteasome degradation; an oxidative stress leads to conformational changes in Nrf2-Keap1 complex that prevent ubiquitylation and allow Nrf2 to translocate to the nucleus, where it forms a heterodimer with sMaf and binds the antioxidant response elements (AREs) in the promoter region of genes involved in redox regulation [35]. The Nrf2-target antioxidant enzymes (AOE) include: proteins of the thioredoxin (TXN)-based system, fundamental in reducing oxidized protein thiols; enzymes involved in heme and iron metabolism, e.g., heme oxygenase-1 (HO-1); in ROS and xenobiotic detoxification, e.g., NAD(P)H quinone oxidoreductase-1 (NQO-1), superoxide dismutase (SOD), catalase (CAT), glutathione peroxidase-1 (GPx) and glutathione S-transferase (GST); in GSH synthesis and regeneration, e.g., glutamate cysteine ligase (GCL) and GSH reductase (GSR), as well as NADPH regeneration (glucose 6 phosphate dehydrogenase, G6PD) [36]. 

The Nrf2-mediated response has been shown to be activated or down-modulated depending on the phase of replicative cycle and type of virus. For example, RSV infection was found to down-regulate Nrf2 expression in airway epithelial cells and consequently AOE-related genes. Indeed, Nrf2 mRNA levels were decreased following RSV infection and the nuclear localization of the protein was decreased in infected cells compared to uninfected ones. The authors suggested that this down-modulation was responsible for the rapidly generated ROS causing lung inflammation and oxidative damage [37]. HIV Tat protein has been shown to induce Nrf2 pathway [38], even though in another study it has been shown that a dysregulation of this pathway increased Tat-induced oxidative burden [39].

Some studies have reported that during the acute phase of HCV infection, infected cells activated the Nrf2/ARE pathway for enhancing the expression of antioxidant genes, and to protect themselves against the HCV-induced oxidative stress [40,41]. Conversely, other studies evidenced a suppression of Nrf2 activation due to viral core and NS3 proteins, causing the delocalization of small Maf proteins from the nucleus and not allowing the formation of active Nrf2/Maf heterodimers [42]. Moreover, the HCV-dependent inhibition of Nrf2-target gene expression is related to the activation of autophagic pathway useful for the viral particles release [43]. Our group evaluated the Nrf2 expression during HCV acute and chronic phases of infection, showing that the protein was down-regulated during early phases of infection, while it was more expressed during the chronic phase, suggesting that the restoration of reducing conditions, through the increasing of the antioxidant response into the cells, could favor viral persistence [17].

Regarding Nrf2 modulation during influenza virus infection, some authors showed that the influenza virus-induced oxidative stress led to Nrf2 nuclear translocation and overexpression of AOE like HO-1, to protect cells from the virus-induced cytopathic effect [44]. Another group found a negative impact of the highly pathogenic influenza virus strains on Nrf2 pathway [45]. Interestingly, in human nasal epithelial Nrf2 knockdown cells, there was an increase of influenza virus entry as well as replication, and the supplementation with Nrf2 activating antioxidants inhibited viral replication [46]. 

Among Nrf2-dependent gene products, there is the G6PD enzyme [36], whose deficiency has been associated to the susceptibility to viral infection. G6PD is the first and the rate-limiting enzyme of the pentose phosphate pathway, which is responsible for the production of reducing equivalents of NADPH, used for regenerating GSH, and it was found to affect ROS production. It has been demonstrated that in G6PD knockdown cells there was an increase of susceptibility to human coronavirus (HCoV) 229E and enterovirus (EV) 71 infections [47,48]. Other studies reported a higher susceptibility to Hepatitis A virus (HAV) and Hepatitis E virus (HEV) infections in G6PD-deficient subjects than in normal individuals [49,50]. Furthermore, our preliminary results showed a strong down-modulation of G6PD enzyme in cells infected with influenza virus (unpublished data). 

All these evidences suggest that virus-induced oxidative stress could be an interesting target for developing effective strategies to control viral infections. 

## 2. Redox-Modulating Agents as Antivirals

### 2.1. Thiol-Based Agents: NAC, GSH and Analogues 

As oxidative stress is linked to several pathological conditions, the use of antioxidants in the treatment of a broad spectrum disorders, including infectious diseases, has become object of several studies that highlighted the potential but also the shortcomings of this kind of therapy [51,52]. The most promising molecules include thiol-based agents, represented by GSH and its precursor N-acetylcysteine (NAC).

NAC is a natural antioxidant found in plants especially of the *Allium* species, whose thiol group directly scavenges ROS. Moreover, it is a precursor of the amino acid cysteine and therefore of GSH [53]. It was recognized as a drug in 1960s and approved by the Food and Drug Administration (FDA) as an antidote in paracetamol overdose/acute hepatic injury and as a mucolytic agent in bronchopulmonary disorders. Other indications include psychiatric disorders. However, there are still controversies about its use as drug or supplement [53]. Regarding viral infections, NAC has been recently shown to be effective in reducing DENV infection in vitro and to improve clinical signs, including liver injury, in DENV-infected mice [54]. Several years earlier, NAC had been already shown to exert a certain protective effect in mice infected with influenza virus, alone [55] or in synergistic combination with the antiviral drug ribavirin [56] and oseltamivir [57]. In another study it has been suggested that NAC reduced influenza virus-induced acute lung injury by inhibiting TLR-4 expression in the lung [58]. In epithelial lung cells infected with influenza A and B viruses and with RSV, NAC was shown to inhibit mucin and pro-inflammatory cytokines production [59]. Other studies questioned NAC anti-influenza activity, limiting its efficacy to specific viral strains [60]. Moreover, at the moment, very limited clinical trials are available to justify the pharmacological use of NAC in respiratory viral infection in vivo.

As said above, intracellular GSH decrease is a common event in viral infections, although with some differences depending on the type of virus, infected cell and host factors, e.g., sex [6], while several in vitro and in vivo studies demonstrate that the administration of GSH inhibits viral replication. In 1995, Palamara et al. provided evidence that exogenous GSH inhibited Herpes simplex virus type 1 (HSV-1) replication by interfering with late phases of viral life cycle [61]. Then similar effects were observed in HIV-infected macrophages [62]. Cai et al. demonstrated that GSH had antiviral activity both in influenza-virus infected epithelial cells and mice [63]. Although promising results were obtained by GSH treatment, high doses of GSH are necessary to achieve a therapeutic value since it is not easily transported into the cells and tissues. To solve this problem some derivatives with hydrophobic chains of different length were synthesized and tested for antiviral activity, among which the N-butanoyl GSH derivative (GSH-C4) resulted the most potent in inhibiting Sendai and HSV-1 replication, without toxic effects [64]. Then, GSH-C4 has been shown to inhibit HSV-1 replication in macrophages as the anti-herpetic drug acyclovir [65]. During influenza virus infection, our group found that the treatment with GSH-C4 interfered with the protein disulfide isomerase (PDI)-mediated maturation of viral HA in the endoplasmic reticulum, thus inhibiting viral replication. The protective effect of GSH-C4 in influenza virus-infected mice was shown too [34]. 

A different boosting GSH molecule is represented by I-152, which is a conjugate of NAC and s-acetyl-β-mercaptoethylamine (cysteamine, MEA), able to release NAC and MEA and increase GSH content. Its antiviral activity was demonstrated in in vitro and in vivo models [66]. I-152 revealed antiviral activity in human monocyte derived macrophages infected with HIV-1/BaL and in a murine model of AIDS, I-152 treatment was effective in reducing proviral DNA content in lymph nodes and spleen resulting in inhibition of the main signs of the disease [67,68]. Regarding the mechanism of action of I-152 as antiviral, we can hypothesize that, by releasing both NAC and MEA, it could interfere with early and late steps of virus life cycle [62,69].

### 2.2. Polyphenols 

Polyphenols are phytochemicals found in plants, cereals and spices but also in beverages such as tea and coffee [70], that are emerging as interesting compounds in the treatment of several diseases. As antioxidant molecules in fact, they may protect cell components against oxidative damage and, in this way, contribute to defense from various degenerative diseases associated to oxidative stress. 

Polyphenols are divided into different classes (phenolic acids, flavonoids, stilbenes and lignans) according to the number of phenol rings that they contain and to the atoms that bind these rings to each other. The most studied group of polyphenols is represented by flavonoids that share a common basic structure consisting of two aromatic rings bound together by three carbon atoms to form an oxygenated heterocycle. On the basis of the heterocycle, flavonoids can be divided into several subclasses: flavones, flavonols, isoflavones, flavanones, flavanols (or catechins), anthocyanins, and their polymers proanthocyanidins [70,71].

The mechanism of action of polyphenols is explained, at least in part, by the so-called biochemical scavenger theory which states the capacity of polyphenols to eliminate free radicals by forming stabilized chemical complex and prevent further reactions [72]. There is also evidence of an additional mechanism by which polyphenols may protect against oxidative stress by producing hydrogen peroxide (H_2_O_2_), which can act as signaling molecule and regulate immune response actions, like cellular growth [72]. 

Several studies report that polyphenols are promising agents against DNA and RNA viruses, often thanks to their capacity to modulate the intracellular redox state. Some plant extracts, rich in polyphenols, and/or single components such as flavonoids and anthocyanins, have been found to possess anti-HSV-1 and antioxidant activities [73,74]. We also evaluated the anti-HSV-1 activity of a polyphenol rich extract from *Solanum melongena*, commonly known as eggplant [75]. The extract inhibited the HSV-1 replication when added after viral adsorption and, since it was able to reduce NOX4 expression during infection, its antiviral activity was probably correlated to its antioxidant properties. Other polyphenol rich extracts have been demonstrated to possess anti-HSV-1 activity: for instance, almond skin extract inhibited HSV-1 replication, by blocking its adsorption to cells [76]. More recently, an extract, as well as polyphenol components, derived from pistachios kernels have been shown to have anti-HSV-1 effect too [77]. Resveratrol (RV), a stilbene derived from a variety of plants -probably the best known is the grapevine- has been suggested to be a good candidate as an anti-HSV nutraceutical agent in an overview of the in vivo and in vitro studies conducted to test RV antiviral effect, even if human studies are still lacking [78]. In 2004, Docherty et al. [79] evaluated the effects of a cream containing RV on skin lesions in HSV-1 infected hairless mice and they observed a significant reduction in lesion formation when they used 25% cream. The same authors demonstrated the effect of RV cream in inhibiting viral replication and reducing vaginal and extravaginal-lesion formation in mice infected with both HSV-1 and HSV-2 [80]. Among RV derivatives, oxyRV (trans-2,4,3′,5′-tetrahydroxystilbene) has also been demonstrated to exert anti-HSV activity in animal models [81]. Among in vitro studies, aimed at investigating the mechanisms underlying the RV antiviral activity, it has been found that RV inhibited NF-κB activation into the nucleus and reduced the transcription of viral genes as well as viral DNA synthesis [82]. Moreover, RV showed antiviral activity against other members of *Herpesviridae* family, as Epstein Barr virus (EBV), by interfering with multiple targets, including suppression of NF-κB pathway [83].

Replication of the Flaviviruses West Nile (WNV), Zika (ZIKV) and DENV was inhibited by the use of Delphinidin and Epigallocatechin Gallate (EGCG, the predominant catechin from green tea), that reduced in particular the infectivity of DENV and ZIKV [84]. Other polyphenols, such as glabranine, quercetin, fisetin and RV possess anti-DENV effect [85,86,87]. Polyphenols from both green and black tea, have been shown to have anti-HCV activity by inhibiting the viral entry into the cells [88,89]. Among bioflavonoids that inhibit HCV in culture models, quercetin has been evaluated in a phase I study, resulting safe in chronic HCV-infected patients [90].

The use of polyphenols is under investigation also for the treatment of retrovirus infections, such as HIV. Indeed, RV treatment has been shown to potentiate the inhibition of reverse transcription by nucleoside analog reverse transcriptase (RT) inhibitors (NRTIs) in PBMCs infected with HIV-1 clinical isolates [91]. RV and pterostilbene blocked HIV-1 replication acting at reverse transcription step [92]. Finally, EGCG showed antiviral activities against HIV-1 infection. These beneficial effects seem to be mediated by increasing nuclear levels of Nrf2 and decreasing levels of NF-kB [93]. 

A great interest in the use of polyphenols and other natural compounds has also emerged against the respiratory viruses. With regard to Coronaviruses (CoVs), to date, there are no licensed vaccines or specific drugs for prevention or treatment of infection and therapy is focused on supportive care to relieve symptoms and in more severe cases to also support function of vital organs [94]. Recently, Lin SC et al. [95] demonstrated that RV inhibits Middle East respiratory syndrome-coronavirus (MERS-CoV) replication by using in vitro model, while other groups found that a library of flavonoids efficiently blocked the enzymatic activity of SARS-CoV 3C-like protease (3CLpro) [96]. Regarding SARS-CoV-2, due to the similar structure with SARS-CoV (more than 82% identity), the same flavonoids effective against viral 3CLpro of MERS and SARS [96] could be also efficient against SARS-CoV-2. 

Lopes BRP et al. [97] reported that the acetylated quercetin, a modified molecule in order to ameliorate its solubility and specificity, interacts with F-protein of *Paramyxoviridae* members to block viral adhesion to infected cells. 

Regarding influenza virus, our group demonstrated that RV can exert anti-influenza effects through a blockade of the nuclear-cytoplasmic translocation of viral ribonucleoproteins (vRNP) and reduced expression of late viral proteins [98]. In this context, RV efficacy against influenza virus replication was not related to glutathione-mediated antioxidant activity [98], but rather to the inhibition of cellular kinases [4]. Furthermore, anti-influenza properties of stilbene and chalcone derivatives have been reported [99].

Computational and molecular studies revealed also a higher affinity of several flavonoids including quercetin for the NA active site of influenza virus [100]. Furthermore, curcumin, a polyphenol derived from turmeric (*Curcuma longa*), widely used as spice and coloring agent in food, added at subcytotoxic doses, greatly reduced the yield of influenza virus by interfering with viral HA activity [101]. However, curcumin shows low bioavailability in vivo. It is unstable and quickly metabolized into derivatives; for this reason, its metabolites and/or analogues have also been tested, showing different degrees of antiviral efficacy [102]. Our group also tested different curcumin analogues against influenza virus, finding that these compounds exerted an antiviral activity mainly affecting intracellular metabolic pathways, including redox-sensitive p38 MAPK, rather than acting directly on viral proteins function [103]. Other authors demonstrated that the inhibition of influenza virus infection by curcumin was due to the activation of the antioxidant Nrf2 pathway and inhibition of virus-induced inflammatory pathways [104]. Recently, several clinical studies on curcumin protective role in different diseases have been reviewed underlying that the problem of bioavailability actually was overcome by higher doses of curcumin without toxic effect or by using it in combination with other compounds, as well as in certain formulations; in this way curcumin could be an effective, safe and cheap nutraceutical [105]. However, in the large number of these trials, those evaluating curcumin effects in infectious diseases are still very few and need to be validated [106].

Sulforaphane (SFN) is an isothiocyanate (sulfur compound) abundant in vegetables, mainly of the cruciferous family. It is known for its cytoprotective effects demonstrated in several in vitro and in vivo studies. SFN, as well as EGCG, has been shown to be a powerful activator of the Nrf2 pathway. The use of SFN and EGCG suppressed influenza virus replication indicating a causal relationship with the induced Nrf2 activation by these molecules [46]. Regarding EGCG and tea catechins, although their anti-influenza activity has been demonstrated long ago, evidences of clinical efficacy of tea consumption is not conclusive [107].

Some authors report that several extracts characterized by different polyphenols show anti-influenza activity, to which all components can contribute. Recently, we tested the antiviral effect of hydroalcoholic extract from female inflorescences of *Humulus lupulus*, an essential ingredient in beer, on influenza virus replication and we found that the extract inhibited viral replication of different virus strains. Furthermore, it was able to restore reducing conditions by increasing GSH content [108]. Ginseng products, used as herb nutritional supplements, are orally consumed and fermented to ginsenoside compounds by the intestinal microbes. The use of fermented ginseng extract developed immunity and protected against infection from different influenza strains in vivo and in vitro models [109]. *Rubus coreanus* is a species of black raspberry native of Korea, Japan and China; it is also rich in polyphenols and possesses the highest antioxidant capacity among fruits and vegetables. A seed extract, that is the left over from the production of wine or juice, has been found to inhibit influenza virus types A and B. In particular, its polyphenol gallic acid was capable of disrupting viral particles [110]. The cranberry extract Oximacro, with a high content in proanthocyanidins, already shown to have anti-HSV activity [111], recently has been shown to inhibit influenza A and B viruses by hampering HA-mediated attachment and entry into the cells [112].

### 2.3. Vitamins and Oligoelements

#### 2.3.1. Vitamins 

Vitamins are essential micronutrients whose deficiency, mainly associated to malnutrition, has been recognized as the cause of important diseases. In the last decades growing evidences have associated vitamin deficiency to infections and subsequent responses [113,114]. Moreover, some vitamins, such as vitamin C and E, have well known antioxidant properties. 

Vitamin C, also known as ascorbic acid, belongs to the group of water-soluble vitamins, those that cannot be accumulated in the body, but must be regularly taken through nutrition. It is contained in fresh fruits and vegetables such as citrus fruits, kiwis, spinach, tomatoes and peppers. Although it is considered a powerful antioxidant, it can also act as a pro-oxidant when it reacts with iron or copper, producing hydroxyl radicals [52]. There are contrasting data about vitamin C efficacy in preventing and reducing the duration of colds and in general towards viral infections [113,115]. Some studies provide data showing that vitamin C deficiency reduces resistance to various microbial agents, while a supply improves it. This could be partially due to its antioxidant potential, but a wider spectrum of immunomodulating activity has been described [113]. Despite its diffuse use in the general population as a tool against viral respiratory infection, in a systematic Cochrane review, Hemila et al. concluded that vitamin C failed to reduce colds, therefore its regular supplementation is not justified [115].

Vitamin E comprises different fat-soluble antioxidant molecules (four tocopherols and four tocotrienols), present in food, especially in vegetable oils. The form required by humans is the α-tocopherol, a powerful peroxyl radical scavenger, thus preventing lipid peroxidation of the membranes. The resulting tocopheroxyl radicals are reduced back by vitamin C or GSH, but they can act also as pro-oxidants [52]. Vitamin E may regulate the activity of several enzymes through redox modulation. It may affect protein translocation and interaction with the membranes, regulating in this way signal transduction. It has been shown to have anti-influenza activity when used in its reduced form [116]. Some studies reported that vitamin E supplementation confers resistance to infections for its immunostimulatory properties, even if often these effects were little or limited to small groups of subjects reviewed in [117].

Although it is not considered an antioxidant, we have to mention also vitamin D for its important role in immunity. The main source of this vitamin, or calcitriol, is represented by skin, where, under the exposure to ultraviolet rays from sunlight, its synthesis starts from a precursor naturally present in humans. The mechanisms through which vitamin D deficiency may contribute to infections development remains poorly understood. Some mechanisms suggested to explain its role in antimicrobial/ antiviral defenses include immunomodulatory functions, with the induction of antimicrobial peptides, activation of autophagy and apoptosis, and a direct effect on viral factors cannot be ruled out [118].

In a systematic review of the Cochrane library, Visser et al. collected the randomized controlled trials from 2010 to 2016 on the supplementation of one or more micronutrients in HIV-infected patients. The main effects that have been evaluated were mortality, morbidity, and disease progression. Multiple micronutrient supplementation has been found to have little or no effect on mortality, on the CD4+ cell count or viral load, and therefore it did not show clinically significant benefits for HIV-infected people [119]. 

#### 2.3.2. Selenium

Selenium is an essential oligoelement whose beneficial properties on human health date back to 1957 when it was demonstrated to have effects on liver necrosis [120]. It is known today that adequate levels of this element are important for several system/tissue functions, including immunity ones. Selenium is contained in a wide variety of foods, including grains, vegetables, fish, meat and dairy products. The daily intake depends on its concentration in food, the amount of food consumed and the chemical form of the element, which influences the bioavailability, the absorption, tissue distribution and body retention. The predominant form of selenium in food is selenomethionine [121]. Once absorbed, selenium can get metabolized into various small molecular weight seleno-compounds, but most of its effects, including those on immunity, are due to its incorporation, in the form of selenocysteine, into selenoproteins. Selenocysteine, the 21st amino acid, is an analogue of cysteine in which the atom of sulfur is substituted by selenium. So far, 25 selenoproteins have been identified in humans, including enzymes playing an essential role in redox regulation such as GPxs, and TXN reductases. The selenoproteome also includes enzymes localized in the endoplasmic reticulum (ER) and involved in protein folding and ER stress response; iodothyronine deiodinases (regulating thyroid hormone activity), and other members whose function is not well known yet [122]. Selenium deficiency is mainly due to malnutrition or poor diet, but it can be also associated to chronic diseases. Selenium supplementation is studied as therapeutic strategy for different types of diseases, including viral infections, because of its redox modulating effects [123]. Furthermore, as mentioned above, it affects the functions of both innate and adaptive immunity, by enhancing both humoral and adaptive cellular responses, including those toward pathogens, most likely through selenoproteins activities [121,123].

Finally, the use of selenium-based nanoparticles could represent an interesting approach in the treatment of influenza. In fact, recently, nanomaterials with peculiar chemical and physical properties have emerged as a promising alternative for virus control and treatment. In this context, some authors presented novel selenium nanoparticles carrying an anti-influenza drug, for instance amantadine (Se@AM) that have been shown to inhibit influenza virus infection and viral-induced apoptosis through inhibition of ROS-mediated signaling pathways [124]. 

## 3. Effects of Redox-Modulating Agents on Antiviral Response and Inflammation

Immune system functions can be regulated by different redox-modulating molecules. For instance, polyphenols are well known immunomodulatory compounds. Their effects can be summarized in terms of the impact on immune cell populations and modulation of cytokines production, with general immunosuppressive and anti-inflammatory effects. 

Important results concern the population of the innate immune cells, that represent the first barrier against pathogens such as viruses. In this regard, it has been shown that the treatment with RV during the differentiation of dendritic cells (DCs), the most effective antigen-presenting cells (APCs), affects their maturation leading to a tolerogenic population [125]. RV does not merely block DC maturation, but rather redirects it to alternative activation by maturation signals. The authors suggest that this could be explained through RV effects on multiple molecular targets, including protein kinases and NF-κb [125]. 

Monocytes and macrophages also play an important role in the innate immune response. Their recruitment is fundamental for the recognition, phagocytosis and clearance of pathogens; furthermore, they are critical, together with the other immunity cells, in the control and resolution of infection, inflammation and tissue injury. In response to environmental stimuli, macrophages show two forms of polarization, the classic/pro-inflammatory/anti-tumor, also called M1 phenotype, and an alternative/anti- inflammatory (M2) phenotype, and are thought to be essential for maintaining the balance between pro-inflammatory cytokines (IL-1β, IL-2, TNFα, IL-6, IL-8, IFN-γ) and anti-inflammatory cytokines (IL-10, IL-4, TGFβ) [126]. Different polyphenols, including RV, repress macrophages activity, which translates into a reduced production of TNF-α, IL 1-β and IL-6, lower expression of cyclooxygenase-2 (COX-2), inducible nitric oxide synthase (iNOS), and other inflammatory mediators [127]. These immunosuppressive effects have consequences on the immune and inflammatory responses to viruses. For instance, it has been shown that RV has therapeutic potential against RSV-induced airway inflammation and hyper-responsiveness [128].

Curcumin presents also important immunomodulatory functions: it can polarize/repolarize macrophages toward the M2 phenotype and curcumin-treated macrophages have been shown to be highly efficient in antigen capture and endocytosis via the mannose receptor [129]. It also shows anti-inflammatory properties, by inhibiting the expression of pro-inflammatory cytokines such as TNF-α and IL-1, adhesion molecules like ICAM-1 (intercellular adhesion molecule-1) and VCAM-1 (vascular cell adhesion molecule-1), effects mediated mainly by downregulation of NF-κB and STAT3 (signal transducer and activator of transcription) pathways [130]. It has been shown that for these properties, curcumin pre-treatment of human genital epithelial cells prevented HIV-induced mucosal disruption [131].

Quercetin was reported to possess immunosuppressive and strong anti-inflammatory capacities for its action on signal-related kinases ERK and JNK as well as on NF-κB [132]. Among different polyphenols tested on DENV-infected macrophages, quercetin, as well as Fisetin, have resulted able to downregulate the production of proinflammatory cytokines [133].

The immune response to respiratory viruses like influenza virus involves innate immune cells in the early stages of infection and in the absence of protective antibodies, provides a robust T cell response, especially against new viral strains. An exaggerated inflammatory response characterizes the main influenza virus-associated complication, as well as the recent COVID-19, that is the pneumonia [134]. Selected antioxidants, in combination with antiviral drugs, can be beneficial in the treatment of such complication [135]. For instance, it has been reported that a tetramer of RV, vitisin A, strongly inhibited RANTES production by influenza virus-infected alveolar epithelial cells, through interference with Akt and STAT1 signaling pathways [136]. 

Curcumin has been shown to relieve the inflammatory response both in vitro and in vivo models through inhibition of NF-κB pathway and to ameliorate influenza virus-induced pneumonia [137]. Other authors reported that curcumin may exert anti-influenza virus activity by inhibiting influenza virus-induced TLR-2/TLR-4, p38/JNK MAPK and NF-κB pathways [104]. 

A formulation of *Camellia sinensis* (green tea, that is rich in cathechins, as said above) has been shown to be safe and effective in preventing influenza and cold symptoms and this effect was related to an induction of gamma delta T cells function [138]. In a further randomized placebo-controlled study, the consumption of cranberry beverage, rich in polyphenols and in particular in proanthocyanidins, was able to reduce symptoms of illness too; the underlying mechanism was confirmed to be an increased proliferation rate of gamma delta T cells in ex vivo experiments [139]. Furthermore, Kalus et al. [140] demonstrated that CYSTUS052^®^, a *Cistus incanus* plant extract, was effective in patients affected by viral infection of upper respiratory tract, more than green tea extract. The authors questioned on its efficacy as anti-inflammatory agent, but since CYSTUS052 had been already shown to inhibit influenza virus when given prior to infection, they suggested its action on blocking viral adsorption onto the target cells [141]. Black elderberry was another dietary supplement shown to effectively reduce upper respiratory symptoms in a recent meta-analysis [142].

Other studies focused on the evaluation of immunomodulatory properties of micronutrients, such as vitamin C, D, E, and selenium, in influenza virus infection. Vitamin C is an essential factor for the production of anti-viral immune response during the early phase of virus infection through the production of type I IFNs [143]. In fact, L-gulono-γ-lactone oxidase (Gulo) deficient mice, which cannot synthesize vitamin C, died earlier after influenza virus infection, showing lower IFNs levels in the lung respect to wild type mice [143]. In the same in vivo model, although no differences in the viral titer were found, lung pathology was greater in vitamin C-deficient male mice [144]. Moreover, a combinatorial treatment of vitamin C with red ginseng, tested in innate immune cells against influenza virus, enhanced the activation of T and NK cells in mice and increased the survival rate [145]. The effect of both vitamin C and E in influenza virus infected mice has been also studied; vitamin E supplementation was able to restore the endogenous levels of the vitamin and reduced those of lipid peroxidation products (respectively decreased and induced during infection); vitamin C had similar but slighter effect on lipid peroxidation; interestingly, the combination of both had the strongest effect, explained by the authors with the capacity of vitamin C to reduce vitamin E tocopheroxyl radical [146]. The same group demonstrated that vitamin E administered with the NA inhibitor oseltamivir to influenza virus infected mice augmented the antiviral effect of the drug in a dose dependent manner [147]. Both influenza virus infection and vitamin D deficiency (VDD) are more common in cold seasons. Individuals with VDD were shown to have a higher risk of influenza virus infection [148]. Vitamin D, known to possess immunomodulatory effects, might affect the immunogenic response to influenza vaccination, in fact some authors reported a lower seroprotection rates of influenza A virus subtype H3N2 (A/H3N2) and B strain in VDD patients compared to individuals with normal vitamin D levels [149]. Consistently, an open, controlled clinical trial performed on 400 infants randomized in two groups, supplemented with high or low doses of vitamin D, showed that the median durations of symptoms were shorter in the first group compared to the low-dose vitamin D group [150].

Selenium deficiency can affect influenza virus infection too. It was reported that Se-deficient mice developed a more severe lung pathology than Se-adequate mice after influenza virus infection, due to an altered inflammatory response; interestingly, the viral genome has been found also modified in Se-deficient animals, leading the authors to suggest that the nutritional status could influence not only the host response to virus, but also the viral mutation rate [151,152]. Further evidence indicated that selenium supplementation reduced mortality in influenza virus infected mice in comparison with Se-deficient mice, improving the response to the infection [153]. However, a clinical trial testing the effects of selenium supplementation on the immune response to influenza vaccine in elderly showed that these effects could be even negative in some cases, being largely dependent to the form and dose of selenium [154]. Another trial conducted several years ago showed that the elderly participants, who had basal low plasma level of selenium and received low-dose supplement of Se together with another oligoelement, zinc, developed a better humoral response after influenza virus vaccination than people of the placebo group, suggesting that it could be of great importance in reducing morbidity from respiratory tract infections [155]. In general dietary selenium supplementation, in particular for groups at risk of Se-deficiency, might be a low-cost and easily available adjuvant in therapy of viral infections, especially those caused by RNA viruses towards which beneficial effects of Se treatment have been reported [156]. 

### Glutathione and Immune Response Regulation

It is known that glutathione can influence immune responses by acting on different levels of the immune regulatory network, including cellular and humoral responses, proliferation and cytokine production. Although it has been reported that GSH levels influence lymphocyte functions, from T cell proliferation to cytotoxic T cell activity [157,158], in recent years major attention has been focused on the role of redox control in APCs since Th1 and Th2 cell population differentiation or CD8+T cell activity are under the control of APCs. For example, it is known that GSH influences several steps on class II MHC pathways of antigen processing and presentation [159]. Moreover, APCs produce T-cell polarizing cytokines determining Th differentiation. For instance, IL-12 and IFN-gamma are the critical cytokines triggering the downstream signaling cascade to develop Th1 cells and several in vitro and in vivo studies have demonstrated that GSH levels in APCs have a crucial role in determining whether Th1 or Th2 cytokine response patterns predominate in immune responses. Several papers have reported that depletion of GSH decreases IL-12 secretion and inhibits Th1-associated cytokine production while GSH increase has just opposite effects [160,161,162].

**Figure 1:**
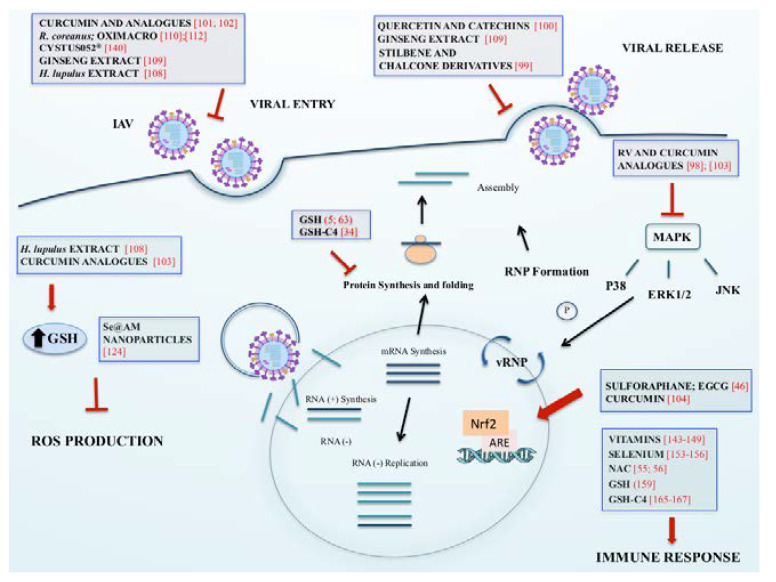
The picture represents the effects exerted by different compounds on influenza virus infection and host response discussed above.

Moreover, the immunoproteasome, that may play an important role during viral infection through regulation of CD8+T cell responses [163], may be affected by redox alterations [164]. Hence, although fewer studies are available about this aspect, we can hypothesize that also the cytotoxic response may be affected by intracellular redox state. Hence, GSH-replenishing molecules may control viral infection through dual mechanisms; on the one hand, they can have a direct effect by modulating specific redox-sensitive pathways exploited by the viruses for their replication, on the other hand they can induce a prevalent Th1 immune response leading to a more robust immune response. Some pro-GSH molecules have demonstrated to modulate the immune response towards antigens and viral infections. Recently, the addition of GSH-C4, by altering the intracellular redox state, was found to modulate the Th1/Th2 balance favoring Th1-type response in old mice infected with influenza virus [165] (Figure 1). Furthermore, GSH-C4 was shown to possess anti-inflammatory properties by inhibiting the NF-κB pathway [166] and reducing redoxins release in LPS-stimulated macrophagic cells [167]. I-152 treatment was found to restore GSH content in spleen and lymph nodes and a balanced Th1/Th2 response in mice infected with LP-BM5 murine leukemia retrovirus [168]. Moreover, both GSH-C4 and I-152 shifted the immune response towards Th1 in mice immunized with ovalbumin or HIV-Tat suggesting that these molecules could also be employed as immunomodulators to enhance existing or to generate new antiviral immune responses [169,170].

These findings indicate that the use of redox-modulating agents for therapeutic purposes is gaining relevance in the treatment of viral infections. Their mechanisms of action can be traced back to their ability to modulate the intracellular redox state by targeting pathways that are exploited by viruses to their advantage. At the same time, by modulating the sensitive redox pathways, they are able to regulate the immune response against viruses. However, the studies are still incomplete. In fact, so far, they are mainly conducted in vitro and in animal models, while for most of the agents, clinical studies are few or discordant. Moreover, regarding phytochemicals, in some studies, the single compounds have been tested, while in others, extracts, therefore a mixture of more compounds that could have additive/synergistic effects, have been used. However, these extracts are not always standardized or correctly titrated [171]. These factors could influence the real in vivo bioavailability, the efficacy and/or the safety in humans. Finally, if the most promising application for these compounds is in combination with antiviral drugs, studies of pharmacokinetic/pharmacodynamic interactions are still lacking too.

## 4. Conclusions

Understanding the complex virus–host interactions, and in particular the redox-regulated intracellular pathways activated and exploited by viruses, represents a novel and promising field for the research of new approaches for the control and treatment of viral infections. This “cell-based antiviral strategy”, aimed at inhibiting cell pathways useful for viral replication could reasonably overcome the serious problem of antiviral resistance that normally arises by using drugs directly targeting viral structures (viral genome or proteins). The induction of oxidative stress, through different mechanisms, is typical of both DNA and RNA viruses, as discussed in this review. In this context, particular attention has been recently devoted to Nrf2 transcription factor that plays a pivotal role in the activation of antioxidant response. Although Nrf2 pathway modulation seems to depend on the phase of viral cycle and type of virus, some studies reported a number of agents effective in activating this pathway and, as a consequence, in restoring intracellular redox balance. These effects are associated to an inhibition of viral replication, as well as to a modulation of the immune and inflammatory responses. In particular, in influenza virus infection, some redox compounds, such as glutathione derivatives and a great number of polyphenols, have been shown to be effective in controlling viral replication and virus-induced inflammation.

In conclusion, even if further studies are needed, especially on the pharmacokinetic/pharmacodynamics aspects, redox compounds can be considered as a promising novel source for developing “cell based” anti-viral agents, able to also modulate immune and inflammatory responses.
